# Impact of time to treatment in first occurrence, non-severe *Clostridioides difficile* infection for elderly patients: are we waiting too long to treat?

**DOI:** 10.1017/ash.2024.46

**Published:** 2024-04-24

**Authors:** Rhett Vandervelde, Mark E. Mlynarek, Mayur Ramesh, Nimish Patel, Michael P. Veve, Benjamin A. August

**Affiliations:** 1 Department of Pharmacy, Henry Ford Hospital, Detroit, MI, USA; 2 Department of Infectious Diseases, Henry Ford Hospital, Detroit, MI, USA; 3 Skaggs School of Pharmacy and Pharmaceutical Sciences, University of California, San Diego, La Jolla, CA, USA; 4 Department of Pharmacy Practice, Eugene Applebaum College of Pharmacy and Health Sciences, Wayne State University, Detroit, MI, USA

## Abstract

**Objective:**

Data evaluating timeliness of antibiotic therapy in *Clostridioides difficile* infections (CDI) are not well established. The study’s purpose was to evaluate the impact of time-to-CDI treatment on disease progression.

**Methods:**

A case–control study was performed among hospitalized patients with CDI from 1/2018 to 2/2022. Inclusion criteria were age ≥65 years, first occurrence, non-severe CDI at symptom onset, and CDI treatment for ≥72 hours. Cases included patients who progressed to severe or fulminant CDI; controls were patients without CDI progression. Time to CDI treatment was evaluated in three ways: a classification and regression tree (CART)-defined threshold, time as a continuous variable, and time as a categorical variable.

**Results:**

272 patients were included; 136 with CDI progression, 136 patients without. The median (IQR) age was 74 (69–81) years, 167 (61%) were women, and 108 (40%) were immunosuppressed. CDI progression patients more commonly were toxin positive (66 [49%] vs 52 [38%], *P* = .087) with hospital-acquired disease (57 [42%] vs 29 [21%], *P* < 0.001). A CART-derived breakpoint for optimal time-to-CDI treatment of 64 hours established early (184, 68%) and delayed treatment (88, 32%). When accounting for confounding variables, delayed CDI treatment was associated with disease progression (adjOR, 4.6; 95%CI, 2.6–8.2); this was observed regardless of how time-to-CDI-active therapy was evaluated (continuous adjOR, 1.02; categorical adjOR, 2.11).

**Conclusion:**

Delayed CDI treatment was associated with disease progression and could represent an important antimicrobial stewardship measure with future evaluation.

## Introduction

*Clostridioides difficile* infection (CDI) remains a significant burden to patients, with an estimated 500,000 cases and 15,000 deaths annually alongside costs exceeding $4.8 billion.^1-3^ Roughly 16.8% of patients develop severe CDI, which is associated with higher morbidity, and factors including elderly age and immunocompromised status are associated with poor outcomes.^3-10^ Despite substantial resources dedicated to identification and treatment, the impact of time-to-CDI-directed antibiotic therapy on patient outcomes has not been characterized and could reflect an untapped opportunity for antimicrobial stewardship.^11^

For other infectious syndromes, national standards of care exist that provide emphasis on prompt antibiotic therapy (i.e., Centers for Medicare and Medicaid Severe Sepsis and Septic Shock Early Management Bundle (SEP-1)).^12,13,14^ To mitigate poor outcomes in sepsis, SEP-1 requires administering antibiotics within three hours of sepsis onset, though this is only satisfied via receipt of intravenous antibiotics. Populations with CDI, in which oral therapy is first-line, are not encompassed by this bundle of care.^15^ During sepsis, a complex cascade involving inflammatory mediators, cellular dysregulation, loss of vascular epithelial integrity, and physiological dysfunction occurs and magnifies in the absence of antibiotic therapy.^16^ CDI pathophysiology is driven by inflammatory effects secondary to toxin production and loss of gut cellular integrity, which can lead to physiological disturbances that are comparable to those seen in sepsis.^17^ In the absence of timely treatment, insults from toxins and caustic enzymes can lead to rapidly progressive illness during CDI.^17^

Various investigations have explored the impact of treatment selection on outcomes, which has historically been emphasized in patient-centered CDI research.^18-20^ Despite similarities with other infectious syndromes in which time to treatment is emphasized and corroborated by better patient outcomes, it remains unknown if timeliness of CDI-directed antibiotic therapy impacts outcomes in patients with CDI.^12^ Given this gap in literature, the purpose of this investigation was to evaluate time-to-CDI-directed antimicrobial therapy in patients with CDI and subsequent impact on progression of disease.

## Methods

### Study design

This was a case-control study conducted at Henry Ford Health (HFH), a five-hospital health-system located in southeast Michigan. Cases included patients who developed CDI progression, controls were patients who did not develop CDI progression. This study was approved by the local institutional review board (IRB #15244) with a waiver of consent.

Patients were included if they were hospitalized at HFH from 1/1/2018 to 2/22/2022, were ≥65 years, had an initial microbiologically confirmed CDI episode, were classified as having non-severe CDI at symptom onset based on national guideline criteria,^15^ and received therapy with vancomycin (oral/rectal) or fidaxomicin for ≥72 hours. Persons ≥65 years of age were selected due to evidence suggesting a higher risk of poor outcomes (i.e., disease progression) when compared to those <65 years of age.^5^ Patients were excluded if they met any criteria for severe or fulminant CDI at disease onset,^15^ had missing or inadequate data related to CDI diagnosis (i.e., laboratory values), had an initial CDI diagnosis from an outside hospital, or received CDI-directed treatment (i.e., oral/rectal vancomycin, fidaxomicin, metronidazole, eravacycline) within 1 week prior to CDI diagnosis. Only patients experiencing their first CDI episode were included.

### Data source

Patient data were manually reviewed and extracted from the electronic health record (EHR) using Microsoft SQL Server Management Studio (Microsoft Corp., Redmond, WA, USA). Data extraction parameters were set to identify patients with a positive *C. difficile* test who were older than 65 years old at time of positive test during the prespecified timeframe. The identified patients were then randomized and screened for inclusion. Data collection included patient demographics (ie, immunosuppressed status), comorbid conditions, *C. difficile* severity, and infection characteristics (ie, community- or hospital-onset, stool counts per day), and CDI treatment selection. Patient outcomes included hospital length of stay, odds of CDI progression, and requirement of colectomy. Data were captured by a single investigator using a standardized electronic case report form.

### Key study definitions

CDI progression was defined as disease progression from non-severe to severe or fulminant disease from initial documentation of symptoms within the EHR, or time zero. *C. difficile* severity (ie, non-severe, severe, fulminant) was classified according to criteria from national guidelines.^15^

*C. difficile* infection characteristics (i.e., serum creatinine [SCr], white blood cell [WBC] count, number of stools per day, fulminant disease clinical indicators) were evaluated from the time of symptom onset to patient discharge or 10 days following initiation of CDI-directed antimicrobial therapy. Patients were classified as experiencing CDI progression if they met any of the following after time zero: (1) WBC >15,000 cells/mm^3^, SCr >1.5 mg/dL (severe CDI), or (2) clinical presentation hypotension or shock with systolic blood pressure <90 mmHg or MAP <65 mmHg, ileus on imaging, or the presence of megacolon on imaging with colon diameter at cecum >12 cm (fulminant CDI).^15^ The primary exposure variable of interest was time-to-CDI treatment, defined as the time from initial CDI symptom documentation onset to CDI treatment, in hours.

*C. difficile* onset was categorized as community-acquired CDI (CA-CDI), or within 48 hours of admission, and hospital-acquired CDI (HA-CDI), or greater than 48 hours after admission. For CA-CDI, onset was defined as the same criteria as HA-CDI, with the addition of including the emergency department triage time if symptoms existed at presentation. For HA-CDI, onset was defined as the time the first symptom occurred (≥3 unformed stools or documentation of loose stools in the medical record) prior to *C. difficile* testing being ordered. Patients were considered immunosuppressed if they met any of the following criteria: history of solid organ transplant, human immunodeficiency virus with a CD4 count <200 cells/mm^3^, or receiving prednisone >20 mg/day for >2 weeks.

Clinical cure was defined as treatment completion with symptom resolution and without disease progression while inpatient or treatment continued outpatient for patients discharged within 10 days of treatment initiation. Other secondary endpoints were hospital length of stay (LOS) and ICU LOS from symptom onset, time from treatment initiation to discharge, and requirement of colectomy. Stool counts were obtained using nursing assessment flowsheets, intake/output logs (including rectal tube and bag), and nursing shift notes.

### Microbiology and C. difficile testing

*C. difficile* testing is performed using a multistep algorithm at HFH. The first step involves using an enzyme immunoassay for *C. difficile* glutamate dehydrogenase antigen and enzyme immunoassay for *C. difficile* toxins A and B. Glutamate dehydrogenase positive and toxin positive results were considered diagnostic of CDI. If there is discordance between the results of glutamate dehydrogenase and enzyme immunoassay, polymerase chain reaction (PCR) amplifying the gene target for toxin B is performed to confirm a positive or negative test and interpreted in clinical context. Only symptomatic patients with a positive *C. difficle* test as outlined above were evaluated.

### Statistical analysis

Descriptive statistics (proportion [%], median [IQR]), were used to describe the patients with CDI progression and those without CDI progression. In bivariate analyses, categorical variables were compared using the Pearson χ^2^ or Fisher’s exact test, and continuous variables were compared using the Mann-Whitney U test. To determine variables independently associated with CDI disease progression, variables associated with case/control status (*P <* .25) in the bivariate analyses were entered into a multivariable logistic regression model. Using a backward, stepwise approach, variables were retained in the final model if they were significant and/or their inclusion/exclusion from the model changed the measure of association for the exposure of interest, time-to-CDI-directed antimicrobial therapy, by more than 10%.^21^ Given the lack of literature specifically examining time-to-CDI-directed antibiotic therapy, the variable was evaluated in three distinct ways. First, we assessed time-to-CDI-directed therapy as a continuous variable and compared the median values between progressors and non-progressors using the Mann–Whitney U test. Second, we analyzed time-to-CDI-directed antimicrobial therapy as a categorical variable (<48 hours, 48–72 hours and >72 hours) and compared this between progressors and non-progressors using a χ^2^ test. Finally, we used classification and regression tree (CART) analyses to identify a threshold at which time-to-CDI-directed antimicrobial therapy was significantly different between those who experienced progression versus lack of progression. This breakpoint served as a marker to determine the threshold of categorizing patients into the “early” CDI treatment or “delayed” CDI treatment groups. For the multivariable regression analyses, we evaluated all three ways of examining time-to-CDI-directed therapy and presented all three in the results. Variables included in the model were restricted to an event-to-variable ratio of 10:1; model fit was assessed using the Hosmer-Lemeshow goodness of fit test. The categorical variables were evaluated for collinearity using the χ^2^ test prior to model inclusion; variable selection was prioritized based on the magnitude of association. All statistics were performed with SPSS Statistics for Windows v.26.0 (IBM, Armonk, NY).

## Results

272 patients were included; 136 experienced CDI progression and 136 did not. Baseline patient characteristics of patients with CDI progression and no CDI progression are described in Table 1. The population consisted primarily of white patients (212, 78%), women (167, 61%), and with a median (IQR) age of 74 (69–81) years. Community-onset CDI was present in 79 (58%) and 107 (79%) patients between those with and without CDI progression, respectively (*P* < 0.001). The proportion of patients who were CDI toxin positive was 66 (49%) and 52 (38%) in those with and without CDI progression (*P* = 0.087). The primary CDI treatment in either group was oral vancomycin (123 [90%] CDI progression vs. 130 [96%] no progression, *P*=0.096). Among patients who experienced CDI progression, the median (IQR) time to progression from initial symptom onset was 48 (23–106) hours.

**Table 1.** Baseline characteristics of patients age ≥65 years with *C. difficile* progression from non-severe to severe/fulminant disease and without *C. difficile* progression

Variable, n (%) or median (IQR)	CDI Progression, n = 136	No CDI Progression, n = 136	*P* value
Age, years	73 (69–81)	74 (68–82)	.721
Female sex	79 (58%)	88 (65%)	.26
Race			
White/Caucasian	103 (76%)	109 (80%)	.38
Black/African American	26 (19%)	25 (18%)	.88
Other	7 (5%)	2 (2%)	.17
BMI (kg/m^2^)	25.4 (22.0–31.1)	25.7 (22.1–29.7)	.80
Baseline SCr (mg/dL)	0.94 (0.74–1.21)	0.87 (0.67–1.1)	.018
Baseline WBC (cells/mm^3^)	9.3 (6.4–11.9)	7.8 (5.9–9.9)	.011
Chronic kidney disease	26 (19%)	21 (15%)	.42
Immunocompromised	59 (43%)	49 (36%)	.22
Hypoalbuminemia	77 (57%)	61 (45%)	.052
Concomitant Medications			
Non CDI-antibiotics	104 (77%)	92 (68%)	.11
PPI	80 (59%)	84 (62%)	.62
H2 Antagonist	34 (25%)	35 (26%)	.89
Antidiarrheal	19 (14%)	19 (14%)	1.0
CDI Onset			
Community	79 (58%)	107 (79%)	<.001
Hospital	57 (42%)	29 (21%)	<.001
Positive *C. difficile* test type			
Toxin	66 (49%)	52 (38%)	.087
PCR	70 (52%)	84 (62%)	.087
Initial CDI treatment			
Vancomycin	130 (96%)	123 (90%)	.096
Fidaxomicin	0	4 (3%)	.122
Multiple agents	6 (4%)	9 (7%)	.43
Time to initial therapy, hours	58 (30–88)	42 (23–55)	<.001

Note. BMI, body mass index; SCr, serum creatinine; WBC, white blood cell; PCR, polymerase chain reaction; CDI, *Clostridioides difficile* infection; PPI, proton pump inhibitor.

### Time to CDI-directed therapy

The median (IQR) time-to-CDI-directed treatment was 58 (30–88) hours for those with disease progression compared to 42 (23–55) hours for those without disease progression (*P <* 0.007). When examined as a categorical variable, the proportion of patients with progression significantly increased as the time-to-CDI-directed antimicrobial therapy increased (<48 h: 39.3%, 48–72 h: 48.2% and >72 h: 73.9%). The CART analyses identified a breakpoint of 64 hours in time to receipt of CDI-directed antimicrobial therapy associated with disease progression. Based on this, there were 184 (67%; 72 [53%] CDI progression, 112 [82%] without CDI progression) patients who received early CDI treatment (<64 hours) and 88 (32%; 64 [47%] CDI progression, 24 [18%] without CDI progression) patients who received delayed CDI treatment (≥64 hours).

Using the results of bivariate analyses and clinical rationale, the following variables were included in a multivariable logistic regression model: toxin-positive *C. difficile* test, delayed CDI treatment, and immunocompromised status (Table 2). Other variables were excluded from the model due to unmet statistical criteria. After adjustment for potential confounders, time-to-CDI-active antimicrobial therapy was significantly associated with CDI progression. This was observed regardless of how the time-to-CDI-active therapy was evaluated (CART-derived breakpoint adjOR: 4.63, continuous adjOR: 1.02, and categorical adjOR: 2.11).

Clinical cure was observed in 252/272 (93%) patients and was less common in patients with CDI progression when compared to those without progression (118 [87%] vs 134 [99%], unAdj OR, 0.1; 95%CI, 0.02–0.43; *P* < .001). There were three (2%) patients with CDI progression that required a colectomy compared to none in patients without progression (*P =* .247). Additionally, the median (IQR) hospital length of stay was observed to be longer in those with CDI progression (13 [9–12] days vs 8 [6–12 days, *P* < .001). Patients who received delayed CDI treatment were also observed to have a prolonged median (IQR) hospital LOS compared to those who received early treatment (15 [10–23] days vs 9 [6–13] days, *P* < .001).

**Table 2.** Variables associated with *C. difficile* progression from non-severe to severe or fulminant disease

Variable	unAdjOR, 95%CI	*P* value	AdjOR, 95%CI	*P* value
Delayed CDI therapy >64 h^‡^	4.1 (2.4–7.2)	<.001	4.6 (2.6–8.2)	<.001
Hypoalbuminemia*	1.61 (0.99–2.6)	.052	N/A	–
Immunocompromised	1.36 (0.84–2.2)	.22	1.5 (0.89–2.5)	.13
Toxin positive *C. difficile* test*	0.66 (0.41–1.1)	.09	0.74 (0.58–0.96)	.025
Community-onset CDI^†^	0.38 (0.22–0.64)	<.001	N/A	–
Vancomycin as an initial CDI treatment	2.3 (0.84–6.2)	.096	N/A	–
Receipt of non-CDI antibiotic	1.6 (0.91–2.65)	.11	N/A	–

Note. CDI, *Clostridiodes difficile* infection; N/A, not applicable.

Hosmer–Lemeshow goodness-of-fit test results: *P* = .377.

‡ Classification and regression tree-derived breakpoint.

* Identified to be colinear.

† Identified to be colinear with early CDI treatment.

### Subgroup of hospital-acquired CDI

To account for differences in patients who received early CDI treatment from increased surveillance for CA-CDI, an *a priori* subgroup of patients with hospital-onset CDI was performed. Among this subgroup of 86 patients, there were 57 (66%) patients who developed CDI progression and 29 (34%) who did not. The median (IQR) time-to-CDI treatment in patients with CDI progression was 76 (53–137) hours compared to 49 (37–72) hours (*P* = .002). The median (IQR) LOS from symptom onset was also prolonged in patients with CDI progression (460.1 [286.4–672.4] vs 361.1 [265.2–512.9], *P* = .14). Clinical cure was observed in 49 (86%) of patients with disease progression and 29 (100%) of patients without disease progression (*P* = .047), and one patient with disease progression required a colectomy.

A separate CART analysis was performed to identify dichotomous breakpoints associated with CDI progression within the HA-CDI subgroup. These results identified a breakpoint of 65 hours; there were 40 (47%) patients who received early CDI treatment <65 hours and 46 (54%) patients who received delayed CDI treatment ≥65 hours. In bivariate analysis, patients who received delayed CDI treatment were more likely to develop progression (unAdjOR, 5.3; 95%CI, 2.0–14.0; *P* < .001).

## Discussion

The primary finding of this study is that time-to-CDI-active treatment was associated with developing disease progression. The stability of this finding was reinforced by analyzing the exposure variable in three distinct ways (continuous, categorical, and CART-derived breakpoint) and observing the same finding each time. Within a subgroup of patients with HA-CDI who receive less rigorous screening than CA-CDI, similar findings were observed in the entire cohort. These data suggest that time to therapy could be an important component of CDI management. Notably, hospital LOS from symptom onset was significantly prolonged in patients with CDI progression and in the subset of patients receiving delayed treatment. Examining potential missed opportunities in value-based care, hospitalization-related costs for CDI range from $3,240 to $11,285, and have been reported as high as $21,448 in some analyses.^22,23^ CDI is associated with considerable clinical and economic consequences. Efforts should be undertaken to identify potential opportunities to improve outcomes and downstream healthcare expenditures such as reducing time to effective antimicrobial therapy. Future research is needed to understand the pharmacoeconomic implications of early vs. delayed CDI-directed therapy.

There are limited data evaluating the association between time-to-CDI-directed therapy and disease progression. While CDI remains a priority for health systems and administrators from a regulatory and patient care perspective, empiric antibiotic therapy is usually not deployed nor are there metrics similar to SEP-1 that focus on CDI.^13,14,24,25^ Treatment timelines outlined by SEP-1 have been associated with significant reductions in mortality and LOS, though questions still exist on the magnitude of effect across different strata of illness severity.^26,27^ Nonetheless, most evidence suggests that in severe infectious syndromes, timeliness of treatment is important to ensure optimal outcomes, and delays may contribute to harm.^13,26,28^ If systems of care fail to promptly recognize and treat CDI, there may be associated consequences for vulnerable patients. Clinicians, health care administrators, and policymakers should consider this potential treatment disparity for patients suffering from CDI. Accordingly, antimicrobial stewardship considerations related to the main study findings can include empiric use of therapy in patients identified as high-risk for disease progression as opposed to the practice of treatment in response to positive *C. difficile* tests. These populations could include elderly and immunocompromised individuals. Institutions should also consider the barriers related to infection control and provision of timely CDI therapy.^29^

There are several limitations to this study. The accuracy of data collected is limited to chart documentation and data available following patient discharge was limited; the study design used is appropriate given the research question. Further, the retrospective determination of CDI onset is challenging and may have introduced classification bias, with no well-accepted criteria to establish disease onset. This classification dilemma is not unique to CDI, however, and interrater reliability concerns have been noted even among disease entities with established diagnostic criteria.^30^ To account for these limitations, a single data abstracter and standardized case report form was used, which also has the potential to introduce other types of bias in studies with one versus multiple abstracters. The CDI classification system may suffer from imprecision as it relates to severity of CDI, and other clinical factors can impact laboratory values (i.e., WBC and SCr) beyond the CDI disease process.^15^ As there is no literature to guide early and delayed treatment stratification, CART analysis was used as a hypothesis-generating approach to distinguish groups by time-to-treatment.^31^ Further validation of the derived CART breakpoints observed in this study is warranted; additional data are needed to identify predictors of early or late treatment, which is outside the scope of this study. The population studied also overestimates the true proportion of patients with *C. difficile* who develop disease progression. There was a higher proportion of patients without disease progression who presented with a PCR-positive *C. difficile* test; this could reflect that some patients were colonized and not infected and may have biased disease progression findings. Multivariable logistic regression analysis was used to account for such confounders; there may be some unmeasured or residual confounders that influenced outcome. Further, given that risk factors for delayed CDI treatment are unknown, specific assessment of confounding is difficult. Institutional policies, testing, treatment, and infection control practices may have changed across the observation period.

## Conclusion

In conclusion, patients who received delayed CDI treatment had significantly greater odds of experiencing disease progression based on Infectious Diseases Society of America classification criteria. In addition, delayed treatment was associated with a higher likelihood of prolonged hospitalization. These findings should be prospectively validated within a larger clinical arena.
